# Development and validation of the Korean version of the Reading the Mind in the Eyes Test

**DOI:** 10.1371/journal.pone.0238309

**Published:** 2020-08-31

**Authors:** Hye-Rin Lee, Gieun Nam, Ji-Won Hur

**Affiliations:** 1 Department of Psychology, Chung-Ang University, Seoul, Republic of Korea; 2 Department of Psychology, Korea University, Seoul, Republic of Korea; University of Leipzig, GERMANY

## Abstract

The Reading the Mind in the Eyes Test (RMET) is one of the most widely used instruments for assessing the ability to recognize emotion. To examine the psychometric properties of the Korean version of the RMET and to explore the possible implications of poor performance on this task, 200 adults aged 19–32 years completed the RMET and the Korean version of the 20-item Toronto Alexithymia Scale (TAS-20K), the cognitive empathy domain of the Korean version of the Interpersonal Reactivity Index (IRI-C), and the Buss-Durkee Hostility Inventory-Aggression (BDHI-A). In the present study, confirmatory factor analyses confirmed that the hypothesized three-factor solution based on three different emotional valences of the items (positive, negative, or neutral) had a good fit to the data. The Korean version of the RMET also showed good test-retest reliability over a 4-week time interval. Convergent validity was also supported by significant correlations with subscales of the TAS-20K, and discriminant validity was identified by nonsignificant associations with IRI-C scores. In addition, no difference was found in RMET performance according to the sex of the photographed individuals or the sex or educational attainment of the participants. Individuals with poor RMET performance were more likely to experience alexithymia and aggression. The current findings will facilitate not only future research on emotion processing but also the assessment of conditions related to the decreased ability to decode emotional stimuli.

## Introduction

Social cognition encompasses a range of cognitive processes that can be clustered in several major domains, including theory of mind (ToM), empathy, social behavior, and social and emotional perception [1]. These domains are partly overlap but are distinguishable in their function, methods of assessment, and brain areas involved [2]. Of these, emotion perception is one of the most heavily researched aspects of social neuroscience [3]. Emotion perception refers to the ability to accurately recognize and interpret emotional expressions in others [4]. It has also become an important issue in the field of mental health since it predicts clinical problems and maladaptive coping styles in individuals [5, 6]

Perhaps the most widely used test to assess the ability to perceive emotions in others is the Reading the Mind in the Eyes Test (RMET) [7, 8]. This four-option multiple-choice test consists of 36 photographs of the eye sections of faces (8 with positive emotional valence, 12 with negative and 16 neutral) [9], and participants are asked to infer the mental state expressed by the eye region only. The RMET was originally intended for assessing deficits in ToM and "mindreading" in individuals with autism spectrum disorder [7]. Currently, as studies of the use of the RMET with different clinical populations (e.g., borderline personality disorder and alexithymia) have accumulated, the RMET is used as a measure of emotion recognition ability rather than ToM [10]. To date, the literature that introduced the revised version of the RMET has been cited more than 5200 times (Google Scholar citing sources, July 2020), and the test has been translated into several languages, including Turkish [11], Swedish [12], Japanese [13], Italian [14], and Brazilian Portuguese [15].

There have also been many studies using the RMET in South Korea [16–19]; however, the RMET has not yet been validated in Korea. In this study, we aimed to verify the Korean version of the RMET and identify its psychometric properties. A secondary goal was to replicate the findings of the effects of demographic features on RMET performance [14, 20]. Thus, we investigated whether RMET profiles varied according to the sex and/or educational attainment of the subjects. Regarding the sex variable, sex-based differences in overall scores, accuracy rates for sex-matched stimuli, and sex-unmatched stimuli were explored as well.

The implications of low RMET performance were also of interest in this study. Growing evidence has indicated that the impaired ability to discriminate emotion correlates to clinical problems such as alexithymia (i.e., incompetence at recognizing one’s own or others' feelings) [21, 22], emotion dysregulation [23], and increased vulnerability to mental disorders [24]. In particular, inaccurate emotion perception appears to play a causal role in externalizing symptoms or aggressive relationship behaviors [25, 26]. A vast body of literature has shown that children, adolescents, and adults who demonstrate less mastery of emotion perception are more likely to reveal their aggression with physical, verbal, delinquent, and bullying behaviors [27–30]. In clinical research on emotion recognition deficits in those who are aggressive toward themselves (e.g., alcohol abusers) or others (e.g., subjects with high fetal testosterone exposure) [31, 32], the RMET has also been used as a primary research tool. However, there is no information available about the relevant externalizing problems expected in those in the general population with low RMET performance. In this study, we aimed to provide a descriptive account of poor performance on the Korean version of the RMET in the general population by contrasting the aggression of subgroups defined according to whether their RMET scores were in the lowest 25^th^ percentile or highest 25^th^ percentile of the distribution.

Hence, the current study has three objectives: (1) to examine the psychometric properties of the Korean version of the RMET; (2) to identify the potential effects of demographic features on task performance; and (3) to present detailed comparisons of the probable aggressive characteristics of good and poor performers according to their RMET scores.

## Materials and methods

### Participants

For this study, volunteers were recruited via online advertising and flyers posted in public places. A total of 200 people participated in the study (111 females). All participants spoke Korean as their native language and were aged from 18 to 32 years old (mean 23.07 ± 2.67; Table 1). Given that as of 2015, 85% of those in their 20s were university students or graduates, a high education level of the participants was likely (Korean Statistical Information Service. National Statistical Office, 2015. Population census: population by sex, age, marital status and basic education statistics by city and county; available from http://kosis.kr/index/index.do). The majority of the participants were undergraduates and graduate students in diverse areas of study (e.g., industrial information systems engineering, media communication studies, business, economics, theology, organic new material and fiber engineering, international logistics, history, and psychology). The planning and implementation of this study complied with the 1964 Declaration of Helsinki and its later amendments or comparable ethical standards. The study was approved by the Institutional Review Board of Chung-Ang University (IRB No. 1041078-201707-BRSP-148-01), and written informed consent was obtained from all subjects. To protect the participants’ privacy, their names and personal information were coded.

**Table 1:** Demographic characteristics of the participants.

Demographic information	*N* = 200
Age (years)	23.07 ± 2.67
Sex (M/F)	89/111
Education Attainment	
High School Graduate	29
Attending University	120
College Graduate	47
Graduate School Graduate	4
Marital Status	
Married	8
Single or Unmarried	192

### Instruments

#### Reading the Mind in the Eyes Test (RMET)

The RMET consists of 36 black-and-white photographs of only the eye region of faces that depict specific mental states. Each picture is normalized to the same size (15 cm × 6 cm) and is presented along with four adjectives, and the subject is asked to select the word that best represents the feelings or intentions of the person in the image [8]. We used 36 pictures from the Asian version of the RMET [13] to exclude the cultural bias associated with the race of the figures.

The adjectives used in the RMET were translated into Korean by two Korean-born but English-trained researchers presently working in Korea. Then, back translation of the preliminary Korean version into English by another bilingual native speaker of English was performed. The results were found to be nearly identical to the original English version, and minor discrepancies were resolved based on consensus discussion to produce a final result that was comparable to the original English version of the RMET. The Korean version of the RMET is available here. A total of 36 photographic stimuli were presented and each response was automatically recorded using Psychopy (http://www.psychopy.org/) [33].

#### Alexithymia: Korean version of the 20-item Toronto Alexithymia Scale (TAS-20K)

Alexithymia, a syndrome involving a marked inability to identify and describe emotions and an impoverished fantasy life, has very heterogeneous constructs [34]. The Toronto Alexithymia Scale (TAS) is the most widely used 20-item self-report questionnaire for measuring the following [35]: difficulty in distinguishing emotions from physical sensations when confirming their emotions and responses to emotional stimuli (TAS 1), difficulty in describing emotions to others (TAS 2) and the presence of concrete, externally oriented thinking or preoccupation with the details of external stimuli (TAS 3) using a 5-point Likert scale. With regard to convergent validity, we hypothesized that the RMET would be negatively correlated with the subscales from the TAS, especially TAS 1, which assesses “difficulties in identifying emotions”. The Korean version of the TAS (TAS-20K) has been shown to have sufficient internal consistency (0.81) [36].

#### Cognitive empathy: The cognitive empathy variable of the Korean version of the Interpersonal Reactivity Index (IRI-C)

The discriminant validity of the Korean version of the RMET was assessed by considering the correlation with instruments assessing a key element of social cognition other than emotion perception. Here, the discriminant validity of the RMET was investigated by calculating the correlations with the cognitive empathy variable of the Interpersonal Reactivity Index (IRI), which is scored using a 5-point Likert scale. The IRI considers empathy to be a multidimensional construct [37] and provides information about both cognitive empathy (2 subscales: perspective-taking, fantasy) and emotional empathy (2 subscales: empathic concern, personal distress).

Cognitive empathy refers to the ability to take another's perspective and to understand others' mental status by using various cognitive processes, such as attention, perspective taking, abstract thinking, and set-shifting, while the fundamental processes of emotional empathy are involved in recognizing others’ emotions, showing responsiveness to others’ suffering, and expressing empathy [38]. Thus, we used only the cognitive empathy domain of the IRI (IRI-C), which included the perspective-taking and fantasy subscales, to avoid the jangle fallacy [39]. The Korean version of the IRI, which was used in this study, has been shown to have acceptable internal consistency (0.80) and test-retest reliability (0.76) [40].

#### Aggression: Buss-Durkee Hostility Inventory–Aggression (BDHI-A)

To provide extended accounts of the use of the RMET, we intended to analyze the potential link between performance on the Korean version of the RMET and aggression in individuals. From the hostility inventory (Buss-Durkee Hostility Inventory, BDHI) by Buss and Durkee [41], Ko [42] constructed an aggression scale (BDHI-A) by extracting only the subscales measuring active aggressiveness. The self-reported BDHI-A, which consists of 21 items scored with a 4-point Likert-type response scale, assesses assault, indirect aggression, and verbal aggression.

### Procedure

The participants were asked to choose which word best described what the figure in the photograph was feeling for each item presented via Psychopy by tapping a button on the keyboard. The subjects were informed that there was no time limit but that they should answer as quickly as possible. One example question was initially presented, and then the 36 test items were presented. Details regarding the photographic stimuli are referenced in the Asian version of the RMET. The total number of correct items was calculated for each participant. Performance feedback was not given during the experiment, but a brief psychological report was provided to those who requested it after all the procedures were completed. The other self-report questionnaires were successively administered on the same day. In addition, for twenty-seven subjects, the RMET was administered once and readministered 1 month after the initial assessment for test-retest analysis.

### Statistical analysis

The Korean version of the RMET scores was calculated using the overall mean scores across all items. Student’s *t*-tests and chi-square tests were applied to compare continuous and categorical variables, respectively. To verify the adequacy of the three-factor solution (positive, negative, and neutral emotions) of the Korean adaptation, as suggested by Harkness et al. [9], confirmatory factor analysis was conducted using AMOS (SPSS. Inc., Chicago, IL, USA). For this analysis, the RMET items were divided into 3 groups according to emotional valence, and multiple goodness-of-fit indices were used: the root mean square error of approximation (RMSEA), the root mean square residual (SRMR), the comparative fit index (CFI), and the ratio of the chi-square to degrees of freedom (*χ*^2^/df). Pearson's correlation was also employed to examine the discriminant and convergent validity of the scale. Test-retest reliability analysis of the Korean version of the RMET was performed with a subsample (*n* = 27) that completed the retest 1 month later. To explore the clinical implications of the RMET scores, we contrasted the psychological features of only those participants who scored in the highest 25^th^ percentile versus those who scored in the lowest 25^th^ percentile on the RMET by conducting one-way multivariate analysis of covariance (MANCOVA), controlling for age and education level. All analyses were carried out using SPSS version 23.0 (SPSS. Inc., Chicago, IL, USA).

## Results

### Accuracy

**Fig 1:**
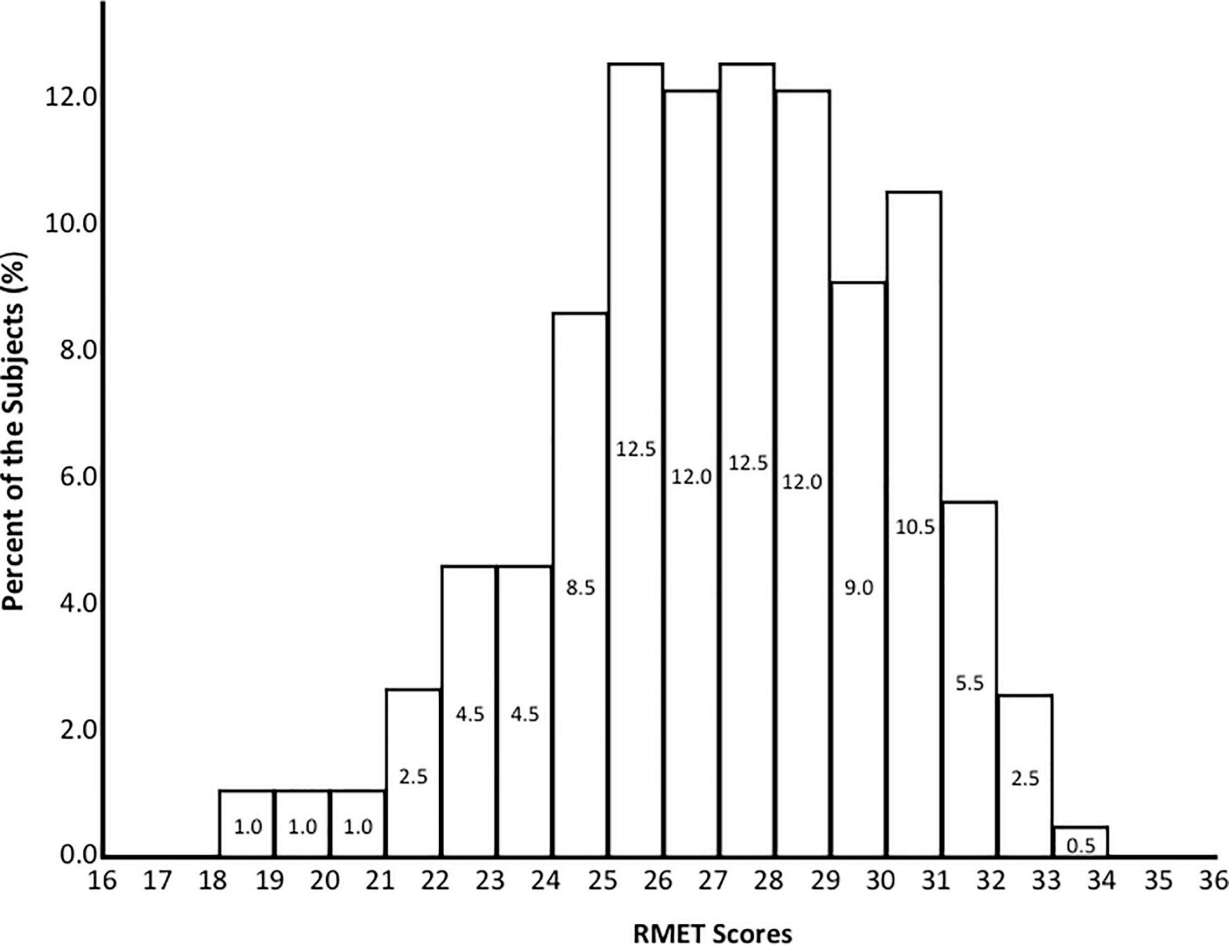
Percentages of participants across RMET scores.

The average RMET score of all participants was 26.57 (SD = 3.01, min: 18, max: 33), and the correct answer rate for the task was 73.81% (Fig 1). The percentages of correct answers for each of the RMET items are shown in Table 2. When comparing the responses to photos of male vs. female figures, the percentage of correct answers was 74.39% for male stimuli and 73.15% for female stimuli (*t*_(199)_ = -1.37, *P* = .174).

**Table 2:** Correct response rate for each item.

No.	S	*%*	No.	S	*%*	No.	S	*%*
**1**	F	*97*	**13**	M	*72*	**25**	F	*98*.*5*
**2**	M	*75*	**14**	F	*64*	**26**	F	*73*.*5*
**3**	F	*63*.*5*	**15**	F	*93*.*5*	**27**	M	*47*.*5*
**4**	F	*82*	**16**	F	*87*.*5*	**28**	F	*46*.*5*
**5**	F	*86*	**17**	F	*86*.*5*	**29**	F	*74*
**6**	F	*53*.*5*	**18**	M	*92*.*5*	**30**	M	*73*
**7**	M	*90*	**19**	F	*74*.*5*	**31**	F	*39*.*5*
**8**	M	*85*	**20**	M	*69*	**32**	M	*88*
**9**	M	*49*	**21**	M	*58*.*5*	**33**	M	*55*.*5*
**10**	M	*54*.*5*	**22**	F	*32*.*5*	**34**	M	*88*
**11**	M	*92*.*5*	**23**	M	*95*.*5*	**35**	M	*63*
**12**	F	*91*	**24**	M	*89*.*5*	**36**	M	*75*.*5*

No., number of stimuli; S, sex of the person in the stimulus photo; M, male; F, female; %, percentage of correct answers per RMET item

### Confirmatory factor analysis

**Fig 2:**
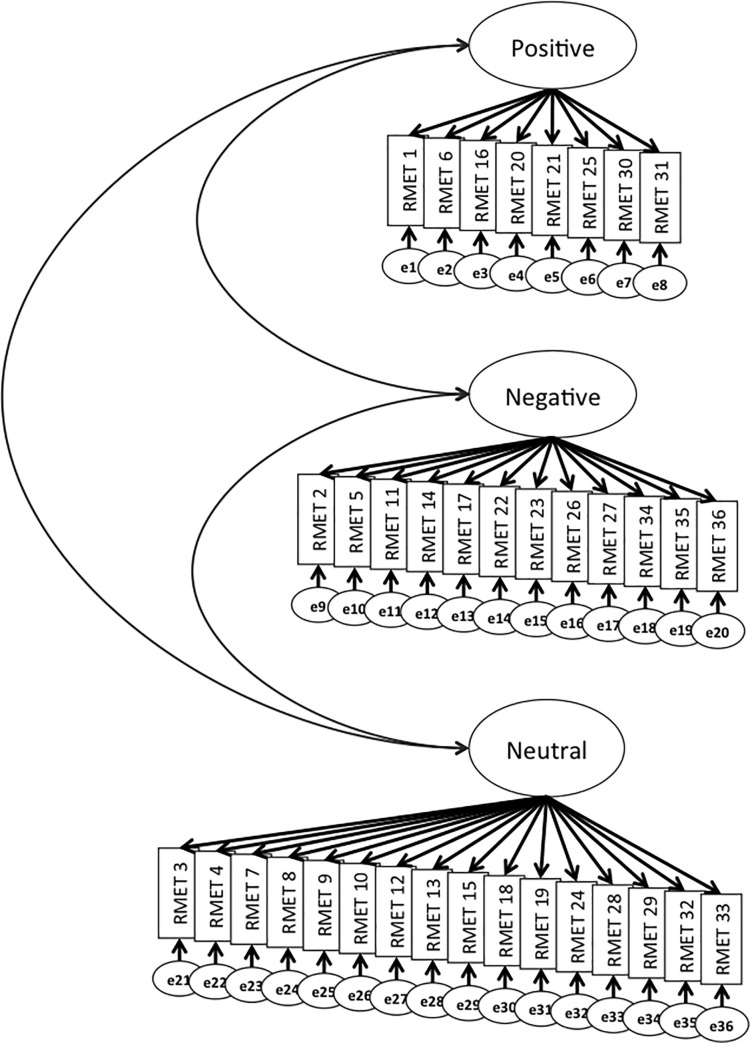
Hypothesized three-factor model of the Korean version of the RMET (n = 200).

The three-factor model of the Korean version of the RMET is depicted in Fig 2. The results of a confirmatory factor analysis showed that the Korean version of the RMET had a good fit to the data. The RMSEA was less than 0.06 (RMSEA = 0.037; 90% confidence interval: 0.028–0.045), the RMR was 0.012 (< .05 indicates good fit), and the RMSEA indicated a marginal fit (GFI = 0.83; between .08 and .09). The ratio of χ^2^ to the number of degrees of freedom (CMIN / df = 1.271; < 3) indicated that the hypothesized model fit the data well. However, as in the prior work on the CFI (> 0.90 indicates good fit) [14], the CFI did not have acceptable values (CFI = 0.45).

### Test–retest reliability

There was no difference between the overall total scores obtained at baseline (mean = 26.48, SD = 3.02) and those obtained at the 1-month follow-up (mean = 26.70, SD = 3.23; *t*_(26)_ = -.51, *P* = .614). There was a significant positive correlation between the test and retest scores (*r* = .74, *P* < .001).

### Validity

The indices of convergent and discriminant validity are presented in Table 3. In support of the convergent validity, the Korean version of the RMET was negatively correlated with the TAS 1 subscale (*r* = -.183, *P* = .010). As anticipated, there was no significant association between the Korean version of the RMET and the IRI-C, indicating discriminant validity (*r* = .060, *P* = .398). On the other hand, the Korean version of the RMET was correlated with the TAS 3 subscale (*r* = -.175, *P* = .013) and the BDHI-A (*r* = -.189, *P* = .008).

**Table 3:** Correlations between RMET, TAS, IRI-C and BDHI-A.

	1	2	3	4	5	6
**1. RMET**						
**2. TAS**	-.176*					
**3. TAS 1**	-.183**	.875**				
**4. TAS 2**	-.053	.842**	.610**			
**5. TAS 3**	-.175*	.553**	.224**	.304**		
**6. IRI-C**	.060	-.218**	-.121	-.188**	-.241**	
**7. BDHI-A**	-.189**	.102	.109	-.006	.146*	-.154*

*P < .05

**P < .01. RMET, Korean version of the Reading the Mind in the Eyes Test; TAS, Korean Version of the 20-item Toronto Alexithymia Scale; TAS 1, difficulty identifying feelings; TAS 2, difficulty describing feelings; TAS 3, externally oriented thinking; IRI-C, cognitive empathy domain of the Interpersonal Reactivity Index; BDHI-A, Buss-Durkee Hostility Inventory–Aggression.

### Demographic variables and RMET performance

#### Sex

To investigate whether sex differences existed in the ability to perceive emotions, a sex-based comparison was conducted. There was no difference in the RMET scores (*t*_(198)_ = .18, *P* = .86 between the male (26.53 ± 2.97, 73.69% correct rate) and female (26.60 ± 3.05, 73.90% correct rate) participants. Additionally, the differences in the correct answers in relation to the sex of the person in the stimulus photo were not significant according to the *t*-tests (male stimulus, *t*_(198)_ = -.08, *P* = .938; female stimulus, *t*_(198)_ = -.20 *P* = .843). When the scores were classified based on the sex of the participant and the sex of the person in the photograph, no disruptions in performance were found when males responded to female photos (72.97% correct rate) or when females responded to male photos (74.44% correct rate). No enhancement in performance was observed for the sex-matched stimuli (74.33% correct rate of males for same-sex photos vs. 73.29% correct rate of females for same-sex photos).

#### Education level

Differences in RMET scores based on the level of education were not statistically significant (*F*_(3,196)_ = 1.21, *P* = .307; 25.62 ± 3.20 for high school graduates vs. 26.75 ± 3.13 for college students vs. 26.74 ± 2.52 for college graduates vs. 26.0 ± 2.71 for graduate students; 71.17% vs 74.31% vs 74.29% vs 72.22% correct rates, respectively).

### Clinical implications of RMET performance

To illustrate the implications of higher or lower RMET performance, MANCOVA analysis was conducted for those who belonged in the highest quartile (the top 25^th^ percentile or 75-100^th^ percentile; scores of 29 or more for the 36 items; *n* = 56) and the lowest quartile (< 25^th^ percentile; scores of 24 or less; *n* = 46) of the RMET score distribution with age and education level as covariances. According to the MANCOVA, there was a significant difference in a series of clinical measures between the highest RMET performance and the lowest RMET performance groups (Wilks’ lambda = .11, *F*_(5,94)_ = 149.50, *P* < .001). In particular, the scores on the TAS 1, TAS 3, and BDHI-A differed depending on the RMET performance level (TAS 1, *F*_(1,98)_ = 5.00, *P* = .028; TAS 3, *F*_(1,98)_ = 8.72, *P* = .004; BDHI-A, *F*_(1,98)_ = 4.31, *P* = .041). The individuals who showed poor RMET performance were more likely to complain of problems involving difficulty identifying feelings, externally oriented thinking and aggression (Table 4).

**Table 4:** MANCOVA to determine the between-subject effects.

		Type III Sum of Squares		*df*		Mean Square	*F*		Sig.
*RMET highest 25% and lowest 25% (covariate*: *age*, *education level)*									
**RMET**	1454.16		1		1454.16		761.15	< .001	
**TAS 1**	187.90		1		187.90		5.00	.028	
**TAS 2**	14.64		1		14.64		0.94	.334	
**TAS 3**	88.39		1		88.39		8.72	.004	
**BDHI-A**	378.14		1		378.14		4.31	.041	

RMET 25, the highest and lowest 25% of scores on the Reading the Mind in the Eyes Tsk; TAS 1, Toronto Alexithymia Scale-difficulty identifying feelings; TAS 2, Toronto Alexithymia Scale-difficulty describing feelings; TAS 3, Toronto Alexithymia Scale-externally oriented thinking; BDHI-A, Buss-Durkee Hostility Inventory Scale—Aggression.

## Discussion

The present results support the use of the Korean version of the RMET as a measure of the ability to recognize emotions. The average RMET score in the Korean general population appears to be 26.57 ± 3.01, and this measure is quite similar to earlier findings from validation studies (Hungary 27.8 ± 5.0; Italy 24.8 ± 4.2; UK 28.3 ± 3.2; USA 27.3 ± 3.7 or 25.7 ± 4.9) [11, 14, 43–45]. Notably, the hypothesized three-factor solution based on three different emotional valences of the items (positive, negative, or neutral) provided excellent goodness-of-fit indices in confirmatory factor analysis. The Korean adaptation was also found to have good test-retest reliability, which is comparable to the results for the existing RMET versions translated into other languages [46].

The findings also indicated good construct validity in terms of convergent and discriminant validity: the TAS-20K (alexithymia) total score and TAS 1 (difficulty identifying feelings) subscore were negatively correlated with the RMET scores, while the IRI-C (cognitive empathy) score, which was expected to be discriminated from the emotion perception measure [1], had no correlation with the scores on the Korean version of the RMET. In terms of empathy, in particular, it is true that there are many shared neural substrates between emotion perception and empathy, but the current evidence from neuroimaging studies elucidates the distinction among social cognition processes such as emotional perception, cognitive empathy, and emotional empathy [38, 47, 48]. The negative findings regarding the link between RMET performance and IRI-C score may be evidence of a double dissociation of emotion perception and (cognitive) empathy.

Group comparison statistical tests were also performed to determine whether sex differences or educational attainment influenced RMET performance. As in prior studies, neither sex differences [44, 45, 49, 50] nor educational differences were found in the RMET scores [51, 52]. These results could be evidence that the RMET is applicable for testing emotion perception abilities regardless of the sex or educational level of subjects; however, regarding previous findings on the significant advantage in RMET performance for females [53] and those with higher educational levels [54], further research should continue to explore the impact of demographic factors on RMET scores.

Another noteworthy piece of evidence indicates that RMET performance has significant clinical implications both in terms of alexithymia and aggression. The results of MANCOVA using age and education level as covariates suggested that there were statistically significant differences in ‘difficulty identifying feelings’ (TAS 1), ‘externally oriented thinking’ (TAS 3) and ‘aggression’ (BDHI) between those who achieved higher RMET scores and those with lower RMET scores, which is in line with prior research (e.g., on physical, verbal, and gestural harm) [55–59]. On the other hand, difficulty describing feelings (TAS 2) was found to be unrelated to RMET performance, presumably because even subjects who have difficulties expressing feelings may relatively easily respond to the forced-choice format of the RMET. Therefore, it is recommended that the RMET not be considered a top priority tool for researchers exploring emotional expression deficits.

Of note, the RMET seemed to provide information about not only individuals’ emotion perception abilities but also the underlying mechanisms of externalizing behaviors. This is supported by previous findings. For instance, individuals with difficulty reading their own or others' emotions may perceive neutral social interactions as hostile [59], and such deficits in emotion perception may lead to subjective anger and aggression [60]. Future follow-up studies should examine the causal effects of an attenuated ability to interpret individuals’ own emotional states on maladaptive behaviors and vice versa.

Several limitations need to be taken into account. Following the previous studies that this study was modeled on, the age range of the subjects in this study was limited to 18–32 years. Moreover, only people living in an urban area were included. Hence, larger samples with diverse sociocultural backgrounds may lead to more generalizable results. The lack of another emotion-related task is a second limitation of this study. Comparisons of multiple measures reflecting real-world social functioning or physiological reactivity in response to emotional stimuli with RMET performance should be considered in the future to allow generalization of the results to a broader population, thus clarifying the clinical implications of RMET performance. Lastly, we only tested the three-factor model of the RMET in this study. Contrasting the different hypothetical models of the Korean version of the RMET might lead to further elucidation of the factor structure of the RMET.

In summary, the Korean version of the RMET is a robust instrument for evaluating individuals’ ability to recognize facial emotions, and it demonstrated good psychometric properties that are comparable to those of other versions of the RMET. The Korean version of the test also proved to be a reliable tool that was not affected by sex or academic background. The Korean version of the RMET may enable the evaluation of individuals’ functioning with regard to emotion processing, which, in turn, could facilitate the understanding of various psychological processes, from emotional issues to behavioral problems, in a variety of clinical and research environments.

## Supporting information

S1 Dataset (XLSX)
